# Luteolin Induces microRNA-132 Expression and Modulates Neurite Outgrowth in PC12 Cells

**DOI:** 10.1371/journal.pone.0043304

**Published:** 2012-08-16

**Authors:** Lian-Fang Lin, Szu-Ping Chiu, Ming-Jiuan Wu, Pei-Yi Chen, Jui-Hung Yen

**Affiliations:** 1 Department of Molecular Biology and Human Genetics, Tzu Chi University, Hualien, Taiwan; 2 Department of Biotechnology, Chia-Nan University of Pharmacy and Science, Tainan, Taiwan; 3 Institute of Medical Science, Tzu Chi University, Hualien, Taiwan; 4 Center of Medical Genetics, Buddhist Tzu Chi General Hospital, Hualien, Taiwan; University of Houston, United States of America

## Abstract

Luteolin (3′,4′,5,7-tetrahydroxyflavone), a food-derived flavonoid, has been reported to exert neurotrophic properties that are associated with its capacity to promote neuronal survival and neurite outgrowth. In this study, we report for the first time that luteolin induces the persistent expression of microRNA-132 (miR-132) in PC12 cells. The correlation between miR-132 knockdown and a decrease in luteolin-mediated neurite outgrowth may indicate a mechanistic link by which miR-132 functions as a mediator for neuritogenesis. Furthermore, we find that luteolin led to the phosphorylation and activation of cAMP response element binding protein (CREB), which is associated with the up-regulation of miR-132 and neurite outgrowth. Moreover, luteolin-induced CREB activation, miR-132 expression and neurite outgrowth were inhibited by adenylate cyclase, protein kinase A (PKA) and MAPK/ERK kinase 1/2 (MEK1/2) inhibitors but not by protein kinase C (PKC) or calcium/calmodulin-dependent protein kinase II (CaMK II) inhibitors. Consistently, we find that luteolin treatment increases ERK phosphorylation and PKA activity in PC12 cells. These results show that luteolin induces the up-regulation of miR-132, which serves as an important regulator for neurotrophic actions, mainly acting through the activation of cAMP/PKA- and ERK-dependent CREB signaling pathways in PC12 cells.

## Introduction

MicroRNAs (miRNAs) are small (19–25 nucleotides) non-coding RNAs that are involved in several biological processes, such as development, morphogenesis, cell proliferation, cell differentiation and apoptosis [1]. Several hundred miRNAs, which act to control the post-transcriptional expression of sets of protein-coding genes and entire pathways, have been identified in mammals and are regarded as prominent regulators for gene expression [2]–[4]. Mature miRNAs are single-stranded RNA molecules that are derived from a immature form of hairpin precursor (pre-miRNA) (approximately 70–100 nucleotides), which are processed from the primary miRNA gene transcripts (pri-miRNA), and usually bind to the complementary sequence in the 3′-untranslated region (3′-UTR) of multiple target genes, which leads to the translational repression or degradation of a target mRNA [5]. Several miRNAs are transcribed and enriched specifically in the mammalian central nervous system (CNS) and may play important regulatory roles in neuronal development and brain function [6]–[8]. Recently, it has been shown that miR-132, one such miRNA that is enriched in the mammalian brain, could be induced by neurotrophic factors and that this could represent a mechanism for fine-tuning protein expression following neurotrophic action [9]. In addition, miR-132 is induced by cyclic AMP (cAMP) response element binding protein (CREB) and is involved in the modulation of dendritic morphology, neurite outgrowth, synaptic plasticity and neuroprotection [10], [11].

Several food-derived phytochemicals are associated with effects that prevent disease including protection from oxidative stress, inflammation, heart disease and cancer [12]. Flavonoids, such as fisetin, epigallocatechin-3-gallate (EGCG), kaempferol, and citrus polymethoxyflavones, have been demonstrated to serve as neurotrophic or neuroprotective agents and to promote neuronal differentiation or to protect neuronal cells against oxidative stress [13]–[17]. Flavonoids could selectively activate a number of neuronal intracellular signaling cascades, most notably the extracellular signal-regulated kinases (ERKs)/mitogen-activated protein kinases (MAPK) and CREB pathways, to regulate the genes involved in neuronal differentiation and survival [18], [19].

Luteolin (3′,4′,5,7-tetrahydroxyflavone, **Figure S1**) is a natural flavonoid that exists in several types of vegetables, fruits, and medicinal herbs and exhibits antioxidant, anti-inflammatory and anti-cancer activities [20]–[22]. Luteolin has been found to possess anti-inflammatory and neuroprotective activities in microglia [23] and to attenuate the neurotoxicity induced by peroxide [24], the neurotoxic agent N-methyl-4-phenyl-pyridinium (MPP^+^) [25] and amyloid β(Aβ)protein [26] *in vitro.* Luteolin can permeate through the blood-brain-barrier (BBB), shows anti-amnesic effects against the toxicity of amyloid (β_25–35_) in mice and attenuates scopolamine-induced amnesia in rats [27], [28]. Luteolin also activates CREB, which is the mechanism underlying its effects on the facilitation of LTP and memory enhancement [29].

**Figure 1:**
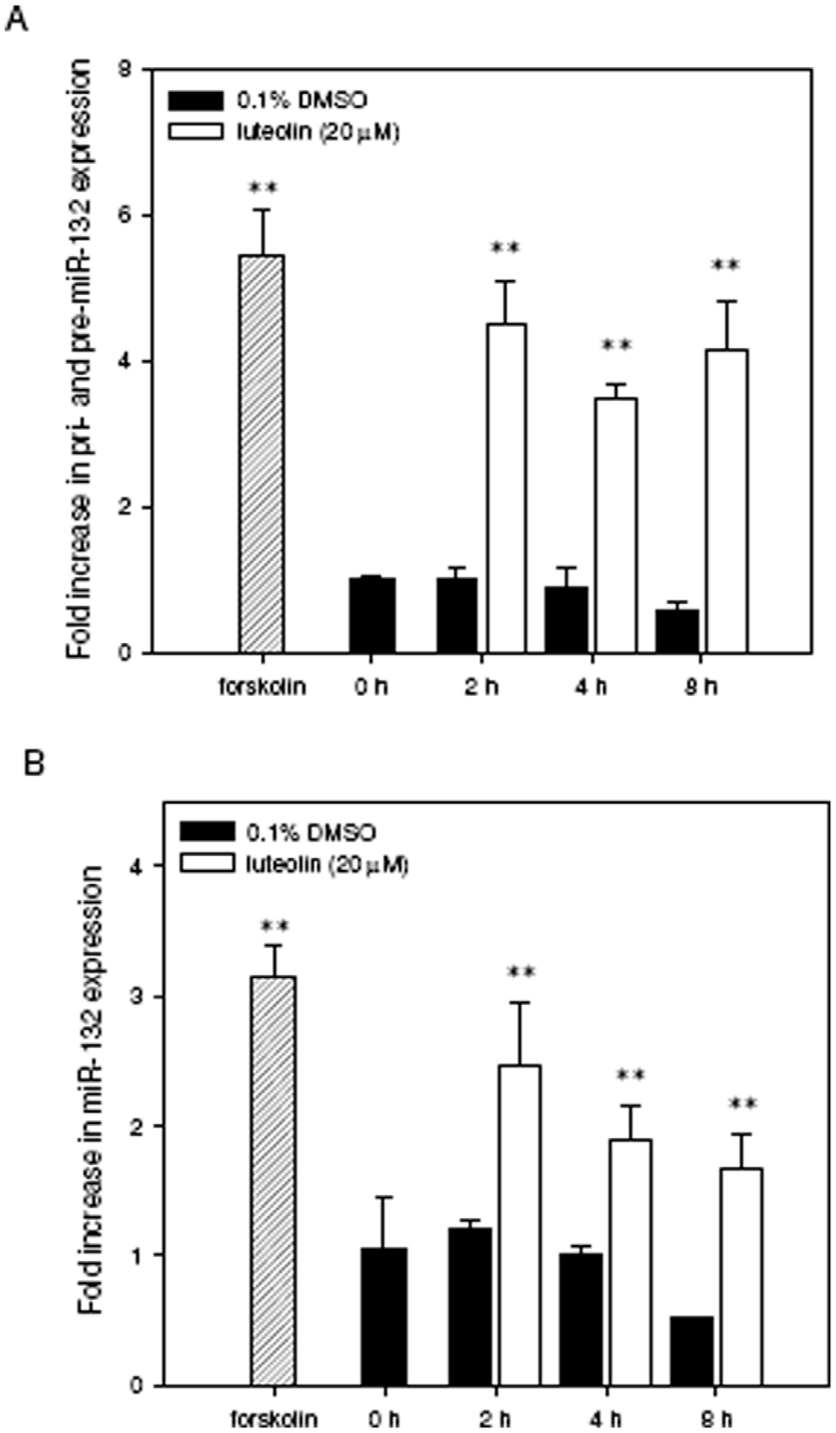
Luteolin induces miR-132 expression in PC12 cells. PC12 cells were seeded on poly-L-lysine-coated 6-well plates in low-serum medium (1% horse serum and 0.5% FBS) for 24 h prior to exposure to forskolin (10 µM) for 2 h, vehicle (0.1% DMSO) or luteolin (20 µM) for an additional 2–8 h. Cellular RNA was then prepared, and the levels of immature miR-132 (pri- and pre-miR-132) (**A**) and mature miR-132 (**B**) were detected by reverse transcription quantitative PCR as described in Materials and Methods. Data represent the mean ± SD from three independent experiments. **p<0.01 represents significant differences compared to vehicle-treated cells.

**Figure 2:**
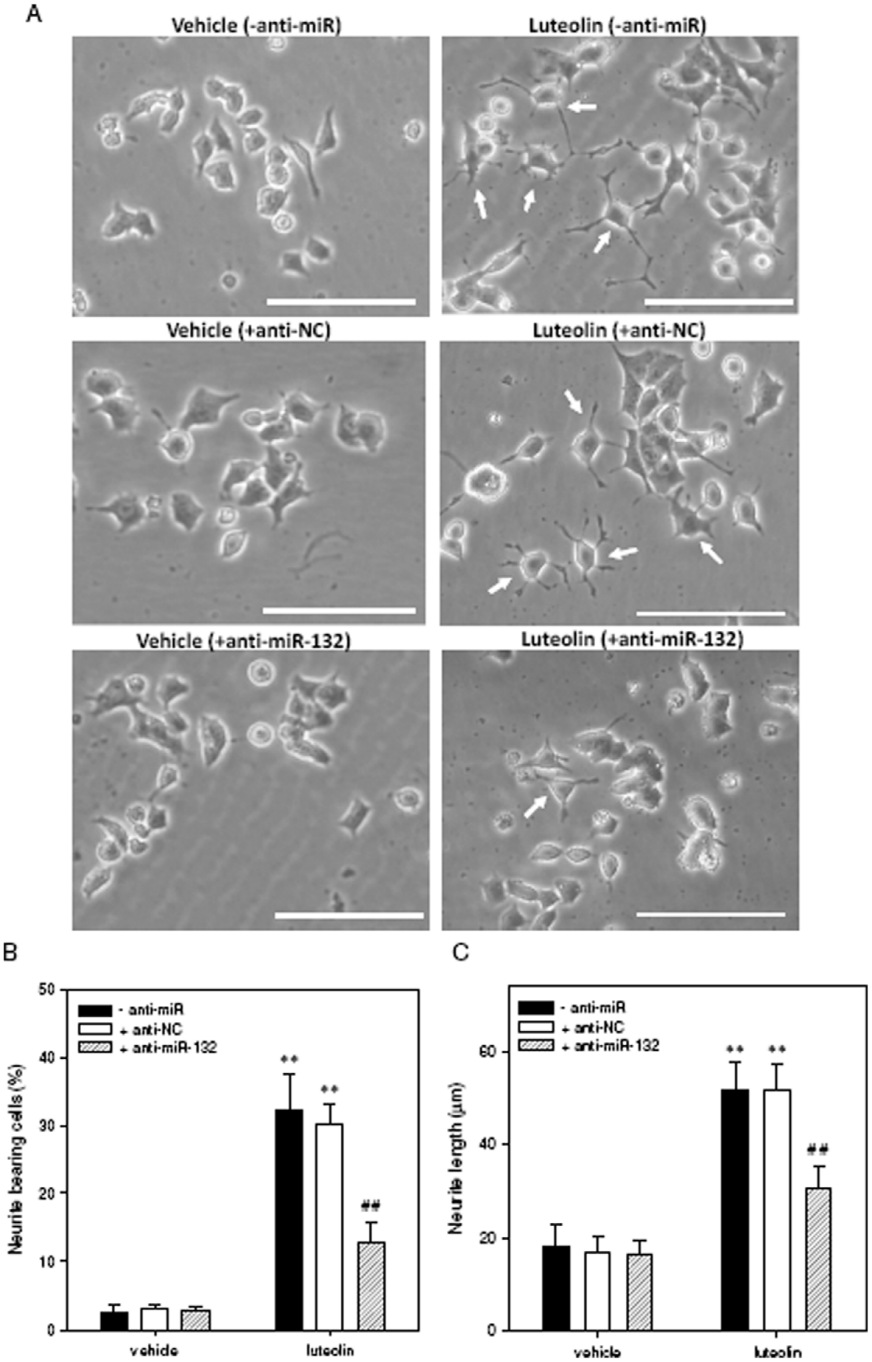
Knockdown of miR-132 expression attenuates luteolin-induced neurite outgrowth in PC12 cells. PC12 cells were seeded on poly-L-lysine-coated 6-well plates in normal-serum medium for 24 h. Cells were then transfected with miR-132 antisense oligonucleotides (anti-miR-132, 150 pmol) or scramble antisense negative control (anti-NC, 150 pmol) for 24 h as described in Materials and Methods. After transfection, the PC12 cells were shifted to low-serum medium (1% horse serum and 0.5% FBS) and exposed to vehicle (0.1% DMSO) or luteolin (20 µM) for an additional 72 h. (**A**) Representative images of neurite outgrowth in PC12 cells. Cell morphology was observed using phase-contrast microscopy and photographed by the digital camera. Arrowheads indicate the neurite-bearing cells. Scale bar, 100 µm. (**B**) Neurite-bearing cells were analyzed as described in Materials and Methods. (**C**) The average maximal neurite length for each of the differentiated cells was analyzed by Image J software. Data represent the mean ± SD from three independent experiments. **p<0.01 represents significant differences compared to vehicle-treated cells. ##*p*<0.01 represents significant differences compared to antisense-untreated group (- anti-miR).

In a previous study, we demonstrated that luteolin is a neurotrophic agent that could promote neurite outgrowth and neuronal differentiation through the activation of the ERK and PKC pathways in PC12 cells [30]. However, there is little information regarding the further details of the molecular mechanism involved in these effects. In the present study, we demonstrate that miR-132 modulates luteolin-mediated neurite outgrowth in PC12 cells. Furthermore, we also explore the possible signaling pathways associated with miR-132 expression that mediate the effect of luteolin on neuronal differentiation.

## Results

### Luteolin Promotes miR-132 Expression in PC12 Cells

To evaluate the effects of luteolin on the expression of miR-132, PC12 cells were cultured in low-serum medium (1% horse serum and 0.5% FBS) and treated with vehicle (0.1% DMSO), forskolin (10 µM; as a positive control) or luteolin (20 µM) for the indicated period. The effect of the luteolin on cell viability in PC12 cell system was also measured by MTT assay as described in Materials and Methods. As shown in **Figure S2**, luteolin sustained cell survival and exerted a slightly proliferative effect in PC12 cells in low serum medium. The levels of immature forms of miR-132 (pri-miR-132 and pre-miR-132) and mature miR-132 were measured by reverse transcription quantitative PCR as described in Materials and Methods. As shown in **Figure 1A and 1B**, treatment of cells with forskolin and luteolin for 2 h significantly increased both the immature and mature forms of miR-132 and the induction remained for 8 h. 20 µM luteolin stimulated immaure and mature forms of miR-132 by 4.5- and 2.5-fold, respectively (*p*<0.01) and the levels were only slightly lower than those of forskolin. This result indicates that luteolin up-regulates miRNA-132 in PC12 cells.

### Effects of miR-132 Up-regulation on Luteolin-mediated Neurite Outgrowth in PC12 Cells

Our previous report had demonstrated that luteolin possessed neurotrophic activity and was able to promote neurite outgrowth in PC12 cells [30]. To investigate the role of miR-132 up-regulation on luteolin-mediated neurite outgrowth in PC12 cells, we used miR-132 antisense (150 pmol) to knockdown the levels of miR-132. **Figure S3A** shows that the cell viability was not affected in the miRNA antisense-transfected PC12 cells. Moreover, we successfully achieved a >95% knockdown of miR-132 in PC12 cells (**Figure S3B**). Furthermore, PC12 cells were transfected with a miRNA antisense negative control or miR-132 antisense and then treated with luteolin (20 µM) for the indicated period; the percentage of neurite-bearing cells and the neurite length were measured as described in Materials and Methods. **Figure 2A and 2B** show that, as expected, luteolin significantly increased neurite outgrowth to 32.3±5.3% relative to vehicle control (2.5±1.2%) (p<0.01). However, the increase due to luteolin treatment in the percentage of neurite-bearing cells was significantly attenuated in those cells transfected with miR-132 antisense (anti-miR-132) (12.8±3.0%) (p<0.01); the increase remained unchanged in antisense negative control (anti-NC)-transfected cells (30.0±3.1%). The average maximal neurite length in luteolin-treated differentiated cells was also significantly reduced by approximately 41% in cells transfected with anti-miR-132 (p<0.01) (**Figure 2C**). To evaluate the effect of miR-132 up-regulation on the neurite outgrowth of PC12 cells, miRNA mimics negative control (miR-NC) and miR-132 RNA mimics (miR-132) were transiently transfected into PC12 cells, and the neurite outgrowth was analyzed as described in Materials and Methods. **Figure S4** reveals that similar to luteolin-mediated phenotypic change, over-expression of miR-132 significantly stimulated neurite outgrowth in PC12 cells. These results show that the miR-132 is involved in the induction of neurite outgrowth in PC12 cells.

**Figure 3:**
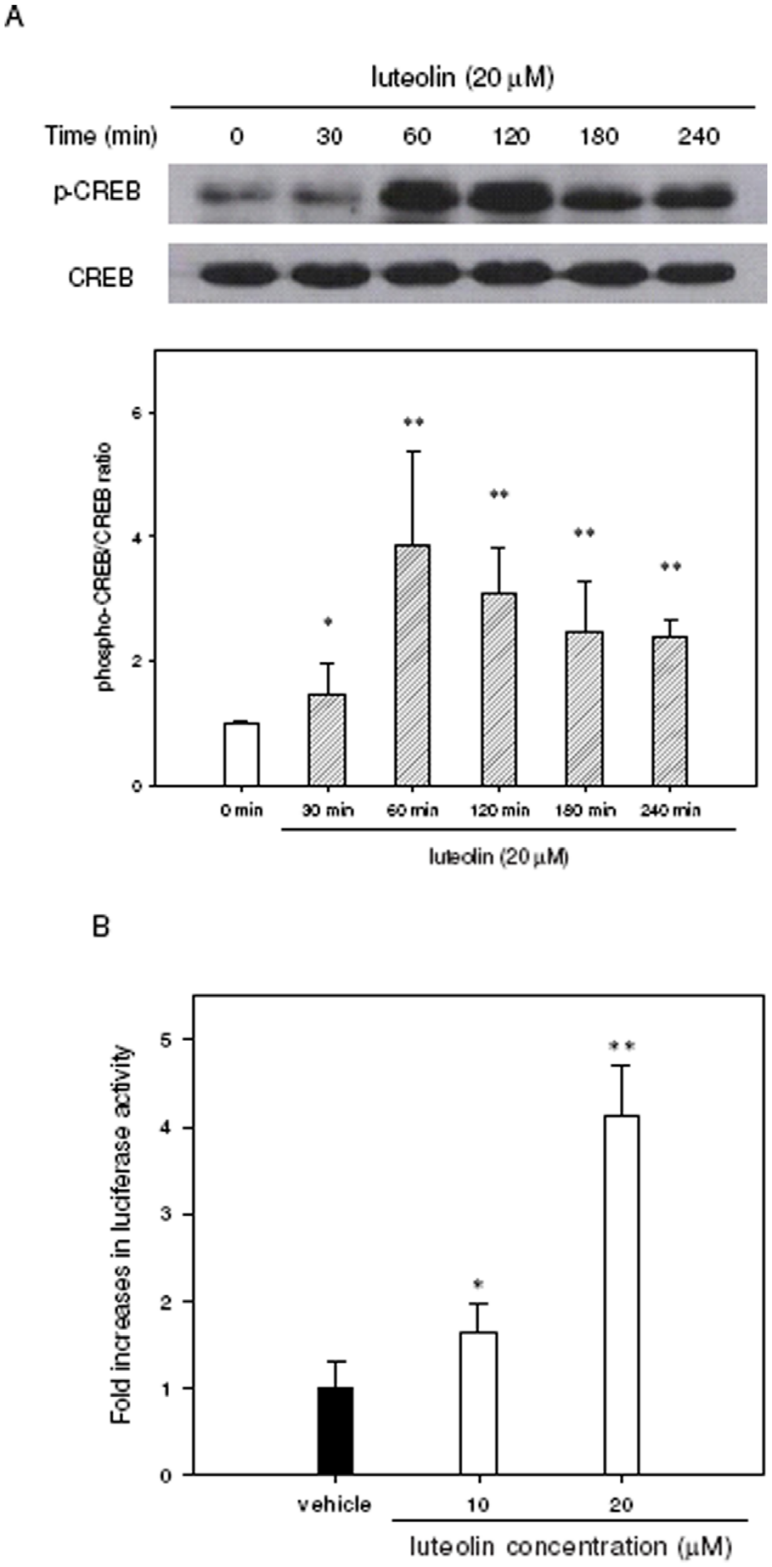
Luteolin increases the phosphorylation and activity of CREB in PC12 cells. (**A**) PC12 cells were seeded on poly-L-lysine-coated 100 mm dishes in normal medium for 24 h and then shifted to low-serum medium (1% HS and 0.5% FBS) for 24 h prior to their exposure to the indicated agents. Adherent PC12 cells were treated with luteolin (20 µM) for 0–240 min. Phospho-CREB-Ser^133^ (p-CREB) and CREB proteins were analyzed by Western blotting as described in Materials and Methods. The immunoblot experiments were replicated at least three times, and a representative blot is shown. The normalized intensity of p-CREB versus CREB is presented as the mean ± SD of three independent experiments. *p<0.05 and **p<0.01 represents significant differences compared to the 0 min group. (**B**) PC12 cells were seeded on poly-L-lysine-coated 24-well plates in DMEM containing 10% horse serum and 5% FBS for 24 h. Cells were then transfected with a CRE-mediated luciferase reporter construct and *Renilla* luciferase control plasmid as described in Materials and Methods. After transfection, PC12 cells were treated with vehicle (0.1% DMSO) or luteolin (10 or 20 µM) for 8 h. Cells were harvested, and the luciferase activities were determined as described in Materials and Methods. The intensities of the luciferase reactions measured in the lysates of the transfectants were normalized to their *Renilla* luciferase control activity. Data represent the mean ± SD from three independent experiments. *p<0.05 and **p<0.01 represent significant differences compared to vehicle-treated cells.

### Effects of CREB Activation on Luteolin-mediated miR-132 Up-regulation in PC12 Cells

It has been shown previously that luteolin facilitates the phosphorylation of CREB (cAMP response element-binding protein) in the hippocampus of normal rats and rescues chronic cerebral hypoperfusion-induced impairment of CREB activation [29]. The phosphorylation of CREB is thought to stimulate the expression of miR-132, which plays critical roles in neuronal differentiation [10], [31], [32]. To investigate whether luteolin-mediated miR-132 up-regulation in PC12 cells also acts through CREB signaling, Western blotting was used as described in Materials and Methods. As shown in **Figure 3A**, the treatment of PC12 cells with 20 µM luteolin increased the phosphorylation of CREB-Ser^133^ over time, peaking in 60 min compared to the 0 min group (*p*<0.01). To evaluate whether luteolin-induced CREB phosphorylation can also activate the transcriptional activity of cAMP response element (CRE), pCRE-luciferase reporter plasmids and *Renilla* internal control vectors were co-transfected into PC12 cells as described in Materials and Methods. **Figure 3B** shows that, when PC12 cells were treated with 20 µM luteolin, the luciferase activities were significantly increased by 4.1-fold compared to those of cells treated with vehicle (*p*<0.01). These results indicate that luteolin induced CREB phosphorylation that, in turn, activated CRE-dependent transcription.

**Figure 4:**
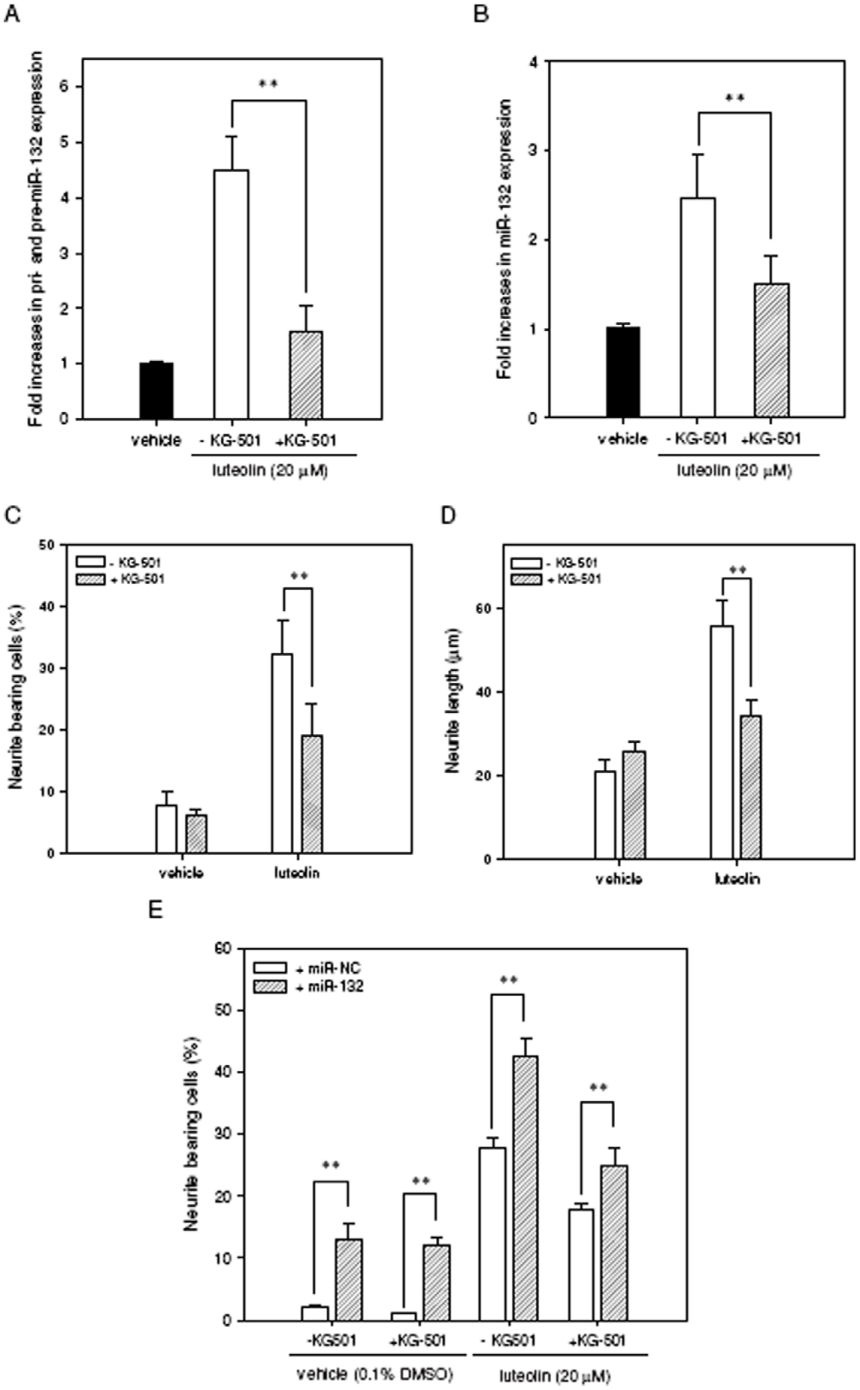
Contribution of CREB activity to miR-132 up-regulation and neurite outgrowth in response to luteolin. PC12 cells were seeded on poly-L-lysine-coated 6-well plates in normal medium for 24 h. Cells were then shifted to low-serum medium (1% horse serum and 0.5% FBS) and were pretreated with the CREB inhibitor KG-501 (10 µM) for 30 min. Cells were then exposed to vehicle (0.1% DMSO) or luteolin (20 µM) for 2 h. Cellular RNA was then prepared, and the levels of immature miR-132 (pri- and pre-miR-132) (**A**) and mature miR-132 (**B**) were detected by reverse transcription quantitative PCR as described in Materials and Methods. Data represent the mean ± SD from three independent experiments. **p<0.01 represents significant differences compared KG-501-non-treated cells. (**C**) PC12 cells were seeded on poly-L-lysine-coated 6-well plates in normal medium for 24 h and then shifted to low-serum medium (1% horse serum and 0.5% FBS) for 24 h prior to exposure to vehicle (0.1% DMSO) or luteolin (20 µM) for an additional 72 h. For the treatment of cells with the inhibitor, adherent cells were pre-incubated with KG-501 (10 µM) for 30 min and then exposed to vehicle (0.1% DMSO) or luteolin (20 µM) for an additional 72 h. Neurite-bearing cells were analyzed as described in Materials and Methods. (**D**) The average maximal neurite length for each of the differentiated cells was analyzed by Image J software. Data represent the mean ± SD from three independent experiments. **p<0.01 represents significant differences compared to the KG-501-non-treated group. (**E**) PC12 cells were transfected with miR-132 mimics (miR-132) or miRNA mimics negative control (miR-NC) for 24 h as described in Materials and Methods. For the treatment of cells with the inhibitor, transfected cells were pre-incubated with KG-501 (10 µM) for 30 min and then exposed to vehicle (0.1% DMSO) or luteolin (20 µM) for an additional 72 h. Neurite-bearing cells were analyzed as described in Materials and Methods. Data represent the mean ± SD from three independent experiments. **p<0.01 represents significant differences compared to miR-NC transfected group.

The direct relationship between miR-132 up-regulation and CREB activation was further investigated by treating PC12 cells with the specific CREB antagonist KG-501 (2-naphthol AS-E phosphate) and CREB siRNA. Luteolin-mediated CRE transcriptional activity was significantly decreased by 10 µM KG-501, which disrupts the CREB:CBP complex and attenuates target gene induction (**Figure S5**). Luteolin-induced immature and mature forms of miR-132 were both markedly attenuated by KG-501 (*p*<0.01) **(** **Figure 4A and 4B** **)**. Furthermore, the treatment of PC12 cells with KG-501 for 72 h also significantly attenuated the percentage of luteolin-induced neurite-bearing cells from 32.3±5.3% to 19.0±5.1% (*p*<0.01) (**Figure 4C**). The average maximal neurite length in those differentiated cells was also reduced by KG-501 treatment (**Figure 4D**).

To further verify the relationship between CREB activation and miR-132-mediated neurite outgrowth, we first over-expressed miR-132 RNA by transiently transfected miR-132 mimics (miR-132) prior to KG-501 treatment and the addition of vehicle or luteolin as described in Materials and Methods. We found that KG-501-caused reduction in neurite outgrowth could be significantly rescued by over-expression of miR-132 in both vehicle- and luteolin-treated cells (**Figure 4E**), indicating miR-132 may function downstream from CREB signaling in PC12 cells.

**Figure 5:**
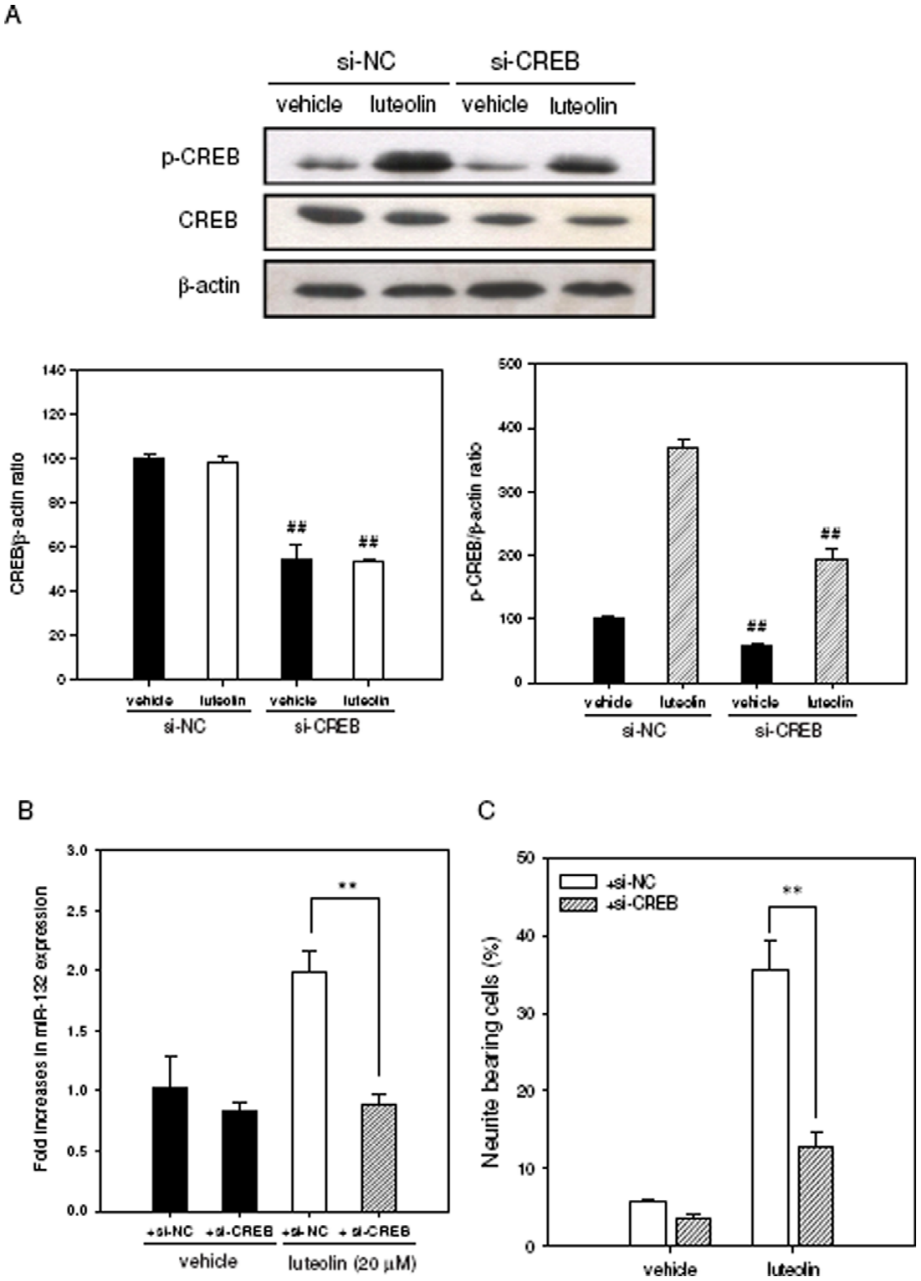
Effects of the CREB protein knockdown on the luteolin-mediated miR-132 induction and neurite outgrowth in PC12 cells. PC12 cells were transfected transiently with siRNA negative control (si-NC) or with CREB-specific siRNA (si-CREB) before vehicle and luteolin (20 µM) treatment. (A) CREB and Phospho-CREB-Ser^133^ (p-CREB) proteins were determined by Western blotting analysis after luteolin treatment for 60 min. β-actin protein is as an internal control. The immunoblot experiments were replicated at least three times, and a representative blot is shown. The normalized intensity of CREB or p-CREB versus β-actin is presented as the mean ± SD of three independent experiments. ##*p*<0.01 represents significant differences compared to the siRNA negative control-transfected group. (B) Effect of CREB knockdown on miR-132 levels after luteolin treatment for 2 h. The levels of miR-132 were determined by RT-Q-PCR as described in Materials and Methods. (C) Effect of CREB knockdown on neurite outgrowth of PC12 cells after luteolin treatment for 72 h. Neurite-bearing cells were analyzed as described in Materials and Methods. Data represent the mean ± SD from three independent experiments. **p<0.01 represents significant differences compared to the siRNA negative control-transfected group.

We next determined the effect of CREB knockdown on the luteolin-induced miR-132 expression and neurite outgrowth by trasnfecting CREB siRNA into cells. We have successfully achieved an ∼50% knockdown of the total CREB as well as phosphorylated CREB proteins in siRNA-transfected cells (si-CREB) as compared with siRNA negative control (si-NC) (**Figure 5A**). Luteolin-induced mature miR-132 (**Figure 5B**) and neurite outgrowth (**Figure 5C**) were significantly reduced in CREB siRNA-transfected cells. These above results suggest that luteolin induces neurite outgrowth through the CREB-dependent up-regulation of miR-132 in PC12 cells.

**Figure 6:**
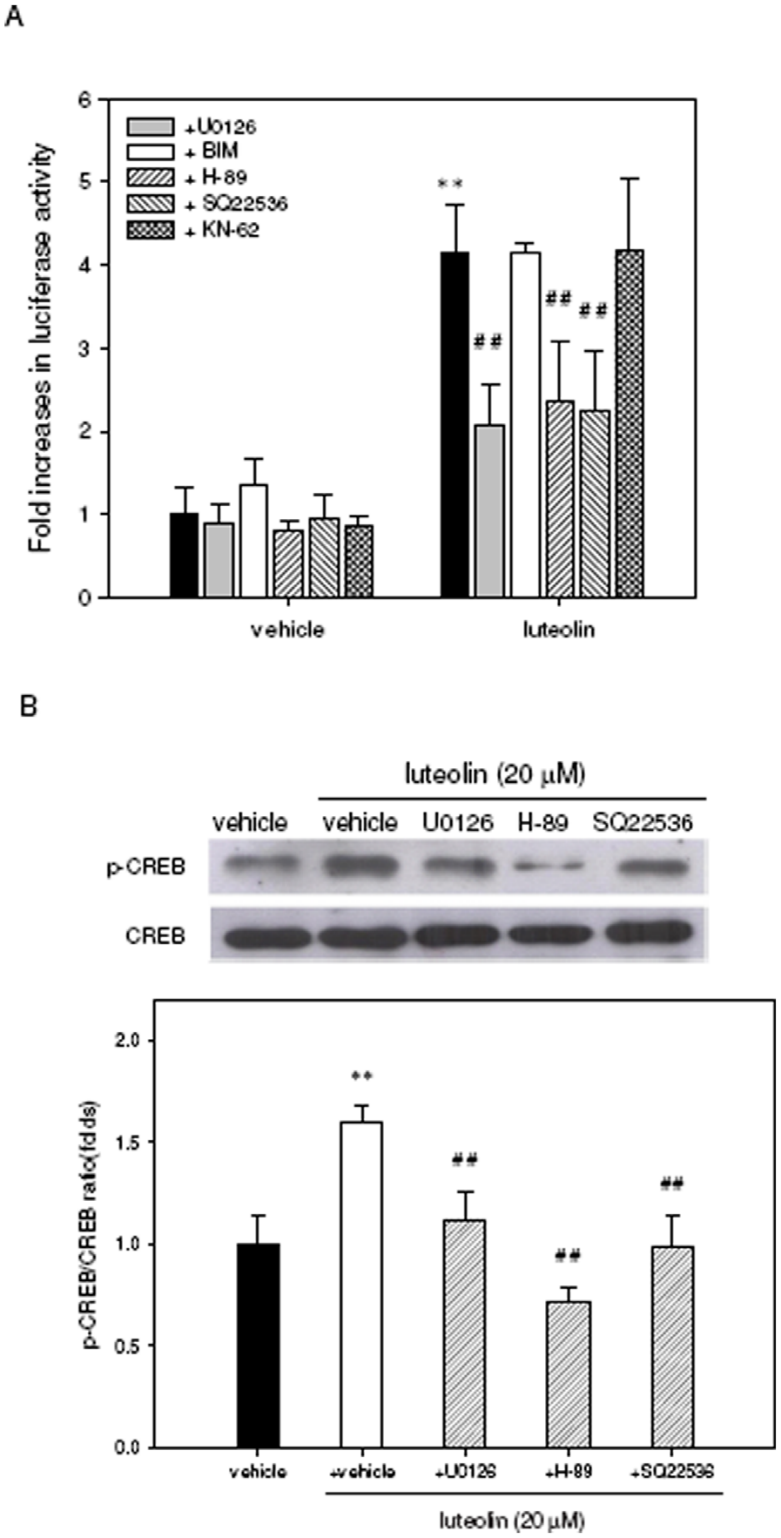
Involvement of ERK, PKC, CaMK and cAMP-dependent PKA signaling in luteoln-mediated CREB activation. (**A**) PC12 cells were seeded on poly-L-lysine-coated 24-well plates in DMEM containing 10% horse serum and 5% FBS for 24 h. Cells were then transfected with a CRE-mediated luciferase reporter construct and *Renilla* luciferase control plasmid as described in Materials and Methods. After transfection, the cells were pre-treated for 30 min with the following inhibitors: 10 µM U0126, 2.5 µM BIM, 10 µM H-89, 500 µM SQ22536, 10 µM KN-62 or vehicle (0.1% DMSO) followed by exposure to luteolin (20 µM) for 8 h. The intensities of the luciferase reactions measured in the lysates of the transfectants were normalized to their *Renilla* luciferase control activity. (**B**) PC12 cells were seeded on poly-L-lysine-coated 100 mm dishes in normal medium for 24 h and then shifted to low-serum medium (1% horse serum and 0.5% FBS) for an additional 24 h of culture. Cells were treated with the inhibitors U0126, H-89 or SQ22536 for 30 min prior to their exposure to vehicle (0.1% DMSO) or luteolin (20 µM) for 60 min. Phospho-CREB-Ser^133^ (p-CREB) and CREB were analyzed by Western blotting as described in Materials and Methods. The immunoblot experiments were replicated at least three times, and a representative blot is shown. The normalized intensity of p-CREB versus CREB is presented as the mean ± SD of three independent experiments. **p<0.01 represents significant differences compared to vehicle-treated cells. ##*p*<0.01 represents significant differences compared to the respective inhibitor-non-treated group.

**Figure 7:**
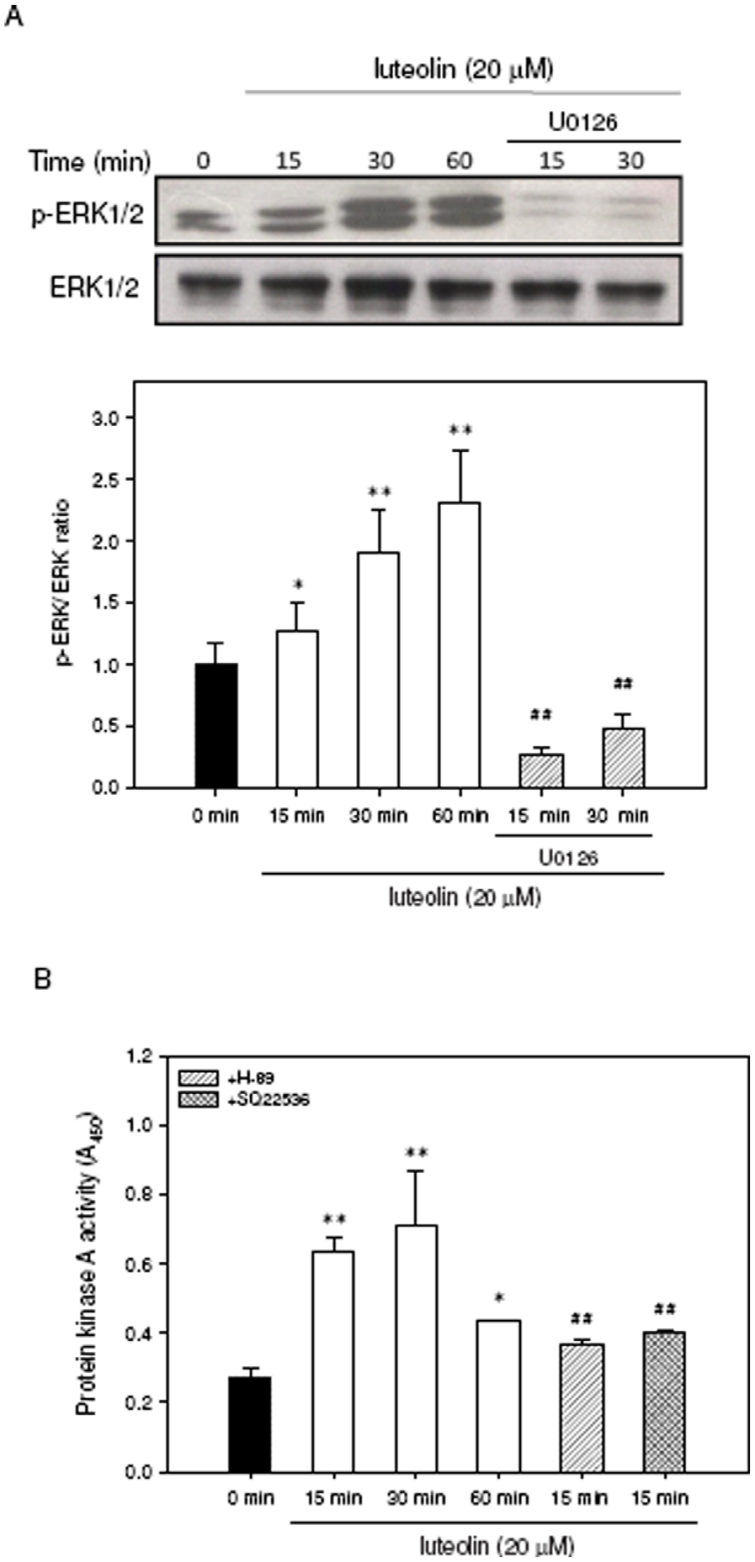
Effects of luteolin on the ERK phosphorylation and PKA activity. PC12 cells were seeded on poly-L-lysine-coated 100 mm dishes in normal medium for 24 h and then shifted to low serum medium (1% HS and 0.5% FBS) for 24 h prior to exposure to indicated agents. (**A**) Cells were treated with luteolin (20 µM) for 0 min, 15 min, 30 min and 60 min. For inhibitor treatment, cells were treated with the inhibitors U0126 for 30 min prior to exposure to luteolin (20 µM) for 15 min and 30 min. Phospho-ERK1/2 (p-ERK1/2) and total ERK1/2 proteins were analyzed by Western blotting as described in Materials and Methods. The experiments were replicated at least three times and a representative blot was shown. (**B**) Cells were incubated with luteolin (20 µM) for indicated period and PKA activity was detected using ELISA kit as described in Materials and Methods. For inhibitor treatment, cells were treated with the inhibitors H-89 (10 µM) and SQ22536 (500 µM) for 30 min prior to exposure to luteolin (20 µM) for 15 min. Data represent the mean ± SD of three independent experiments. *p<0.05 and **p<0.01 represents significant differences compared with 0 min group. ##*p*<0.01 represents significant differences compared to the respective inhibitor-non-treated group.

**Figure 8:**
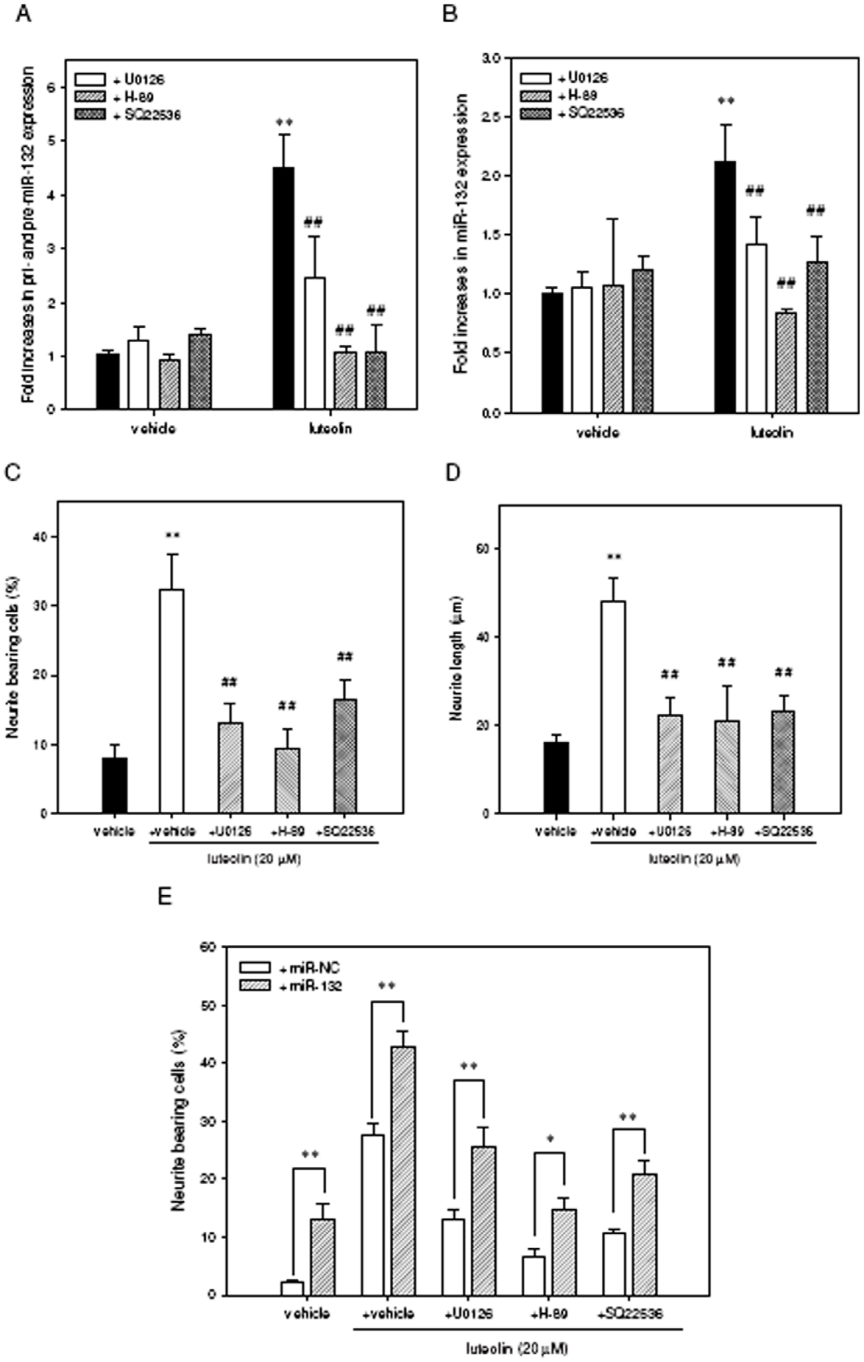
Contribution of ERK- and cAMP-dependent PKA signaling pathways to luteolin–induced miR-132 up-regulation and neurite outgrowth in PC12 cells. PC12 cells were seeded on poly-L-lysine-coated 6-well plates in normal medium for 24 h. Cells were then shifted to low-serum medium (1% horse serum and 0.5% FBS) and then pretreated with the inhibitors U0126 (10 µM), H-89 (10 µM) or SQ22536 (500 µM) for 30 min followed by exposure to vehicle or luteolin (20 µM) for 2 h. Cellular RNA was then prepared, and the levels of immature miR-132 (pri- and pre-miR-132) (A) and mature miR-132 (B) was detected by reverse transcription quantitative PCR as described in Materials and Methods. Data represent the mean ± SD from three independent experiments. **p<0.01 represents significant differences compared to vehicle-treated cells. ##*p*<0.01 represents significant differences compared to the respective inhibitor-non-treated group. (C) PC12 cells were seeded on poly-L-lysine-coated 6-well plates in normal medium for 24 h. Cells were then shifted to low-serum medium (1% horse serum and 0.5% FBS) for 24 h and then pre-treated for 30 min with the indicated inhibitors followed by exposure to vehicle or luteolin (20 µM) for 72 h. Neurite-bearing cells were analyzed as described in the Materials and Methods. (D) The average maximal neurite length for each of the differentiated cells was analyzed by Image J software. Data represent the mean ± SD from three independent experiments. **p<0.01 represents significant differences compared to vehicle-treated cells. ##*p*<0.01 represents significant differences compared to the respective inhibitor-non-treated group. (E) PC12 cells were transfected with miR-132 mimics (miR-132) or miRNA mimics negative control (miR-NC) for 24 h as described in Materials and Methods. For the treatment of cells with the inhibitor, transfected cells were pre-incubated with indicated inhibitors for 30 min and then exposed to luteolin (20 µM) for an additional 72 h. Neurite-bearing cells were analyzed as described in Materials and Methods. Data represent the mean ± SD from three independent experiments. *p<0.05 and **p<0.01 represent significant differences compared to miR-NC transfected group.

**Figure 9:**
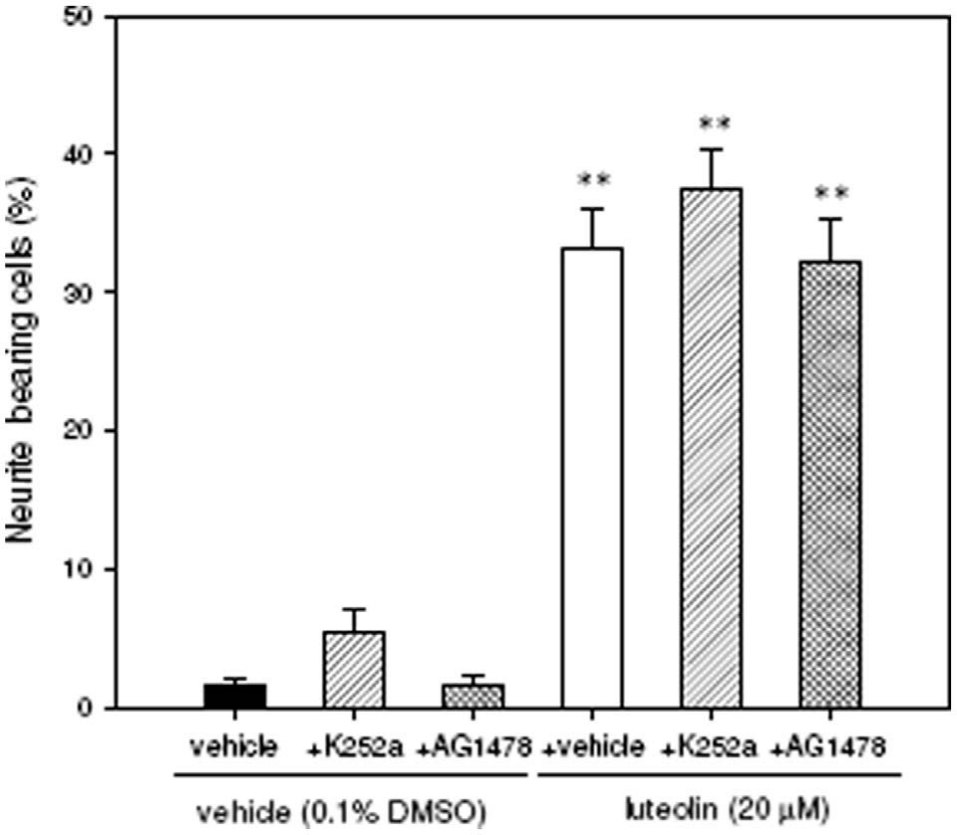
Effects of TrkA and EGFR signaling pathways on the luteolin-induced neurite outgrowth in PC12 cells. PC12 cells were seeded on poly-L-lysine-coated 6-well plates in normal medium for 24 h. Cells were then shifted to low-serum medium (1% horse serum and 0.5% FBS) for 24 h and then pre-treated for 30 min with the TrkA antagonist K252a (100 nM) or EGFR inhibitor AG1478 (2 µM) and then exposed to vehicle (0.1% DMSO) or luteolin (20 µM) for an additional 72 h. Neurite-bearing cells were analyzed as described in Materials and Methods. Data represent the mean ± SD from three independent experiments. **p<0.01 represents significant differences compared to vehicle-treated cells.

**Figure 10:**
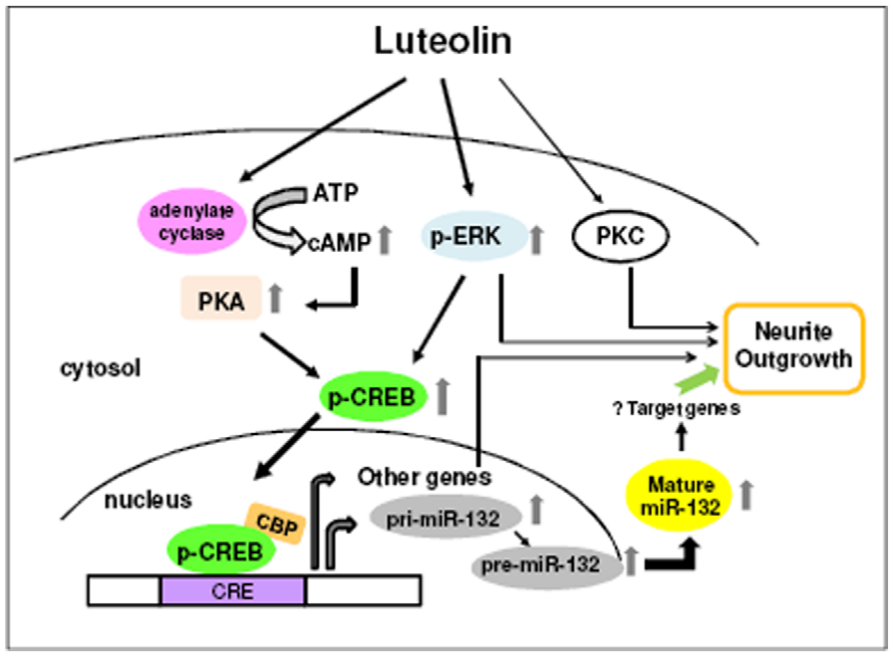
Hypothetical mechanism of luteolin mediation of neurite outgrowth in PC12 cells. Luteolin induces the up-regulation of miR-132, which may serve as a mediator for neurite outgrowth through the activation of cAMP/PKA- and ERK-dependent CREB signaling pathways in PC12 cells. In addition, ERK- or PKC-dependent but CREB/miR-132-independent pathways may also partially contribute to the mediation of neurite outgrowth by luteolin in PC12 cells.

### Signaling Pathways Involved in the Luteolin Mediation of miR-132 Up-regulation and Neurite Outgrowth in PC12 Cells

Many studies support the hypothesis that the transcriptional regulation of CREB represents a converging point for several signaling pathways that are known to regulate a variety of genes involved in neuronal functions [33]–[35]. Thus, the possible involvement of ERK, PKC, CaMK and cAMP-dependent PKA signaling in the mediation of CREB activation and miR-132 up-regulation by luteolin was investigated by utilizing the molecular inhibitors of those pathways. PC12 cells were transfected with reporter plasmids and treated with kinase-specific inhibitors, including inhibitors of MEK1/2 (U0126; 10 µM), PKC (bisindolylmaleimide I, BIM; 2.5 µM), PKA (H-89; 10 µM), adenylate cyclase (SQ22536; 500 µM) and CaMK II (KN-62; 10 µM), for 30 min followed by incubation with 20 µM luteolin before analyzing the luciferase activity. As shown in **Figure 6A**, luteolin-mediated CREB transcriptional activity was markedly attenuated by the inhibitors U0126, H-89 and SQ22536 (*p*<0.01). Furthermore, Western blotting shows that the inhibition of kinase activity by U0126, H-89 and SQ22536 significantly abolished luteolin-mediated CREB phosphorylation (**Figure 6B**). These results indicate that luteolin enhances CREB activation in PC12 cells via MAPK/ERK- and cAMP-dependent PKA pathways.

The effect of luteolin on the ERK activation was further measured using Western blot analysis as described in Materials and Methods. As shown in **Figure 7A**, luteolin increased ERK1/2 phosphorylation after 15 min treatment and remained elevated up to 60 min as compared with 0 min group. This result is consistent with our previous report [30]. The luteolin-induced ERK activation could be significantly abolished by MEK1/2 inhibitor, U0126 (10 µM). Moreover, we analyzed the effect of luteolin on the protein kinase A (PKA) activity using ELISA-based assay kit as described in Materials and Methods. **Figure 7B** shows that PKA activity peaked at 15 min after luteolin treatment (*p*<0.01) and the enhanced PKA activity could be significantly attenuated by co-treatment with H-89 (10 µM) and SQ22536 (500 µM) (*p*<0.01).

We further study whether these above pathways contributed to induction of miR-132 and neurite outgrowth. As shown in **Figure 8A and 8B**, exposure of cells to U0126, H-89 and SQ22536 significantly decreased both immature and mature forms of miR-132 (*p*<0.01). In addition, the treatment of PC12 cells with the inhibitors U0126, H-89 and SQ22536 also attenuated the percentage of neurite bearing cells from 32.3±5.3% to 13.2±2.8%, 9.3±3.0% and 16.4±2.9%, respectively (*p*<0.01) (**Figure 8C**). The maximal neurite length in those neurite-bearing cells was also reduced from 48.1±5.3 µm to 22.5±4.0 µm, 21.1±8.0 µm and 23.2±3.5 µm, respectively (*p*<0.01) (**Figure 8D**). Moreover, the kinase inhibitor-mediated inhibitory effect on neurite outgrowth could be significantly compromised by over-expression of miR-132 (**Figure 8E**). These results confirm critical roles for the ERK- and cAMP/PKA-dependent CREB pathways in the luteolin-mediated up-regulation of miR-132 and enhancement of neurite outgrowth in PC12 cells.

### Luteolin–mediated Neurite Outgrowth is not through Tyrosine Kinase Receptor (TrkA) or Epidermal Growth Factor Receptor (EGFR) Signaling in PC12 Cells

It has been reported that growth factors such as NGF and EGF could act on kinase receptor to promote neurite outgrowth and neuronal differentiation via activation of MAPK/ERK and CREB pathways [36], [37]. It is also known that PC12 cells express functional NGF tyrosine kinase receptor-A (TrkA) and EGF receptor (EGFR) [38]. We further investigate whether TrkA or EGFR signaling pathway is involved in luteolin-mediated neuritogenic functions. **Figure 9** shows that priming PC12 cells with TrkA antagonist K252a (100 nM) or EGFR inhibitor AG1478 (2 µM) did not reverse luteolin-induced effects in PC12 cell differentiation. These data suggest that luteolin promotes neurite outgrowth in PC12 cells via a TrkA- and EGFR-independent signaling pathway.

## Discussion

It has become evident that dietary flavonoids may work as therapeutic agents possessing high neurotrophic activity and that they may exert many effects on cell function within the brain [39]. Recently, several reports have demonstrated that flavonoids can induce neurogenesis and stimulate neuronal regeneration through their interactions with neuronal intracellular signaling pathways and molecules that are pivotal in the control of neuronal differentiation and survival [19], [40]. The flavonoid luteolin, which is abundant in celery, green pepper, parsley, chamomile tea and medicinal herbs, exerts prominent antioxidant and anti-inflammatory activities [41]. Luteolin has also been demonstrated to be an orally available and blood-brain-barrier-permeable compound in animal experiments with promising therapeutic benefits for neurodegenerative disorders [28]. We have previously shown that luteolin promotes neurite outgrowth and maintains neuronal cell survival primarily via the ERK signaling pathway [30]. The major finding of this study is that luteolin increases the levels of miR-132 and promotes PC12 cell differentiation primarily via the cAMP-dependent PKA and MAPK/ERK signaling pathways, which further enhance the phosphorylation of the transcription factor CREB in PC12 cells (**Figure 10**).

The post-transcriptional regulation of gene expression plays critical roles in neuronal development and in the function of the central nervous system (CNS). The transcriptional up-regulation of miRNA, translational regulatory RNA molecules, is a potential mechanism by which signaling transduction cascades could control their cellular functions. The miR-132 is enriched in brain tissue and has been reported to be responsive to neurotrophin signaling, suggesting prominent roles in neuronal morphogenesis, dendritic plasticity and neuronal differentiation [10], [42], [43]. In the current study, we demonstrated that luteolin (20 µM) significantly and rapidly stimulated the levels of mature miR-132 in PC12 cells. The knockdown of miR-132 by antisense RNA oligonucleotides decreased the percentage and the average maximal length of neurite extensions that were induced by luteolin. On the other hand, over-expression of miR-132 significantly enhanced the percentage of neurite outgrowth in PC12 cells. Our findings indicate that luteolin-induced neurite outgrowth occurs, at least in part, through the up-regulation of miR-132 expression. Moreover, since miR-132 is conserved in mammals, we also studied the effect of luteolin on the level of mature form of miR-132 in human neuroblastoma cell line, SH-SY5Y. We found that the level of mature miR-132 peaked at 4 h after luetolin (20 µM) treatment (∼1.5-fold) and sustained for 8 h (∼1.4-fold) as compared with 0 h group (data not shown). This data imply that luteolin up-regulates miR-132 in human neuronal cells to a less extent than in PC12 cells.

The transcription factor cAMP response element binding protein (CREB) is one of several transcription factors that have been demonstrated to be key regulators of gene expression or protein synthesis important for neuronal function. It has been suggested that the phosphorylation of CREB at Ser^133^ controls its ability to regulate transcription that is associated with neuronal differentiation, synaptic function, dendritic spine density and synaptic plasticity [40], [44]–[46]. Recently, the discovery of miRNA that are regulated by CREB suggests the possibility that some of the changes in protein expression may be mediated by miRNA [9]–[11], [47]. Impey et al. identified several miRNAs, including miRNA-132, which are induced by CREB-mediated transcription using an unbiased genome-wide screen in vitro [31]. Vo et al. showed that CREB could bind to the miR-212/132 locus and might regulate the transcription of miRNA-132 [9]. In addition, the ablation of miR-132 has similar effects to the loss of CREB activity on blocking neuronal function, suggesting that CREB signaling is involved in miR-132 expression [43], [48], [49]. In the current study, we clearly show that the treatment of PC12 cells with luteolin enhanced the levels of phosphorylated CREB (Ser^133^) and CRE-dependent transcriptional activity. The addition of the CREB:CBP antagonist, 2-naphthol AS-E phosphate (KG-501) or knockdown of CREB by siRNA markedly attenuated luteolin-dependent induction of miR-132 and neurite outgrowth in PC12 cells. These findings imply that luteolin induces neurite outgrowth through CREB activation, which in turn up-regulates the downstream target, miR-132.

It has been known that the activation of signal transduction pathways, including NGF/TrkA, EGF/EGFR, cAMP-dependent protein kinase A (PKA), MAPK/ERK, protein kinase C (PKC), and calcium-calmodulin kinase II/IV (CaMKII/IV), could converge at the level of CREB signaling, and these signaling pathways have been linked to the control of gene expression and protein synthesis in long term potentiation (LTP), neuronal differentiation, synaptic plasticity and memory [14], [36], [37], [50], [51]. In this study, we find that inhibitors of adenylate cyclase (SQ22536), PKA (H-89) and MAPK/ERK kinase (MEK1/2; U0126) significantly blocked the potentiation of luteolin-induced CREB phosphorylation, CREB activity and miRNA-132 up-regulation. The addition of the adenylate cyclase or PKA inhibitors to luteolin-treated PC12 cells markedly attenuated miR-132 expression to basal levels. This result indicates that the cAMP-dependent PKA signaling pathway plays a critical role in the induction of miRNA-132 expression by luteolin. It has been reported that adenylate cyclase activators such as forskolin promote CREB phosphorylation and induce miR-132 expression in cultured primary rat neurons [52]. It is unclear that the mechanism by which luteolin affects adenylate cyclase activity. Whether it is through the activation of adenylate cyclase alone or in combination with the inhibition of phosphodiesterases, and which catalyze the hydrolysis of cAMP and thereby elevate cAMP concentration to stimulate PKA activity, remains an open question.

In the current study, we find that luteolin induces both immature and mature forms of miR-132 up-regulation and promotes neurite outgrowth via ERK-dependent CREB pathway. Recently, Remenyi et al. has been reported that both pri-miR-132 and pri-miR-212 are encoded by a single non-coding gene and up-regulated via the activation of ERK-dependent CREB pathway [42]. The miR-212/132 cluster was found to produce four miRNAs including miR-132, miR-132*, miR-212 and miR-212*; however, only the function of miR-132 has been studied in neurons. Whether miR-212 is up-regulated by luteolin and also involved in the neuritogenesis of PC12 cells remains to be investigated. On the other hand, in comparison, the addition of MEK1/2 inhibitor only partially inhibited the induction of miR-132 expression by luteolin but potently decreased CREB phosphorylation and completely blocked neurite outgrowth in PC12 cells. It has been reported that neurotrophins such as brain-derived neurotrophic factor (BDNF) induced miR-132 transcription in mouse primary cortical neurons through the ERK1/2 pathway [42]. Previous studies also suggested critical roles for both ERK1/2 and miR-132 in the regulation of neuronal morphology [9], [10], [53]. The data imply that the full behavior of ERK activation is complex, as it may channel through both CREB-miRNA-132-dependent and independent signaling pathways in PC12 cells. In contrast, the PKC inhibitor (BIM) and CaMKII/IV inhibitor (KN-62) did not significantly change luteolin-mediated CREB activation, although the PKC pathway was found previously to be partially involved in luteolin-induced neurite outgrowth [30]. This result shows the involvement of a PKC-dependent but CREB-independent pathway in the induction of neurite outgrowth by luteolin in PC12 cells. In addition, our findings show that the TrkA antagonist and EGFR inhibitor, K252a and AG1478, did not alter luteolin-induced neurite outgrowth in PC12 cells. This result indicates that luteolin induced PC12 cell neurite outgrowth through ERK/CREB pathway which is not associated with TrkA or EGFR activation.

MicroRNAs are known to down-regulate the expression of large numbers of target genes in vivo and to potentially affect large-scale changes in proteomes to direct cellular processes [2], [54]. Several miR-132 targets, including p250GAP, methyl CpG-binding protein 2 (MeCP2), p120RasGAP and p300, have been explored as possible mediators of cellular functions [43], [52], [55], [56]. The role of p250GAP has been reported to regulate spine morphogenesis, neuritogenesis and dendritic plasticity [9], [10], [48], [53]. The role of MeCP2 is rather complex: in addition to affecting the acetylation levels of the entire chromatin complement, it also serves as a CREB activator [52]. To test whether luteolin-induced miR-132 could block expression of p250GAP, we have transfected a luciferase reporter plasmid that contained a p250GAP miRNA response element in the 3′-UTR into PC12 cells and analyzed the effect of luteolin on reporter gene activity. However, reduction of the luciferase activity was not detected at different time points (0, 2, 4, 8 and 16 h) in the luteolin-treated PC12 cells (data not shown). At this stage, we do not know what the downstream target genes of luteolin-induced miR-132 are or how they affect cellular neurogenesis in PC12 cells. Recently, it was reported that miR-132 regulates the differentiation of dopamine neurons by directly targeting Nurr1 expression [57]. The role of Nurr1 on miR-132-mediated PC12 differentiation remains to be investigated.

In conclusion, our current results demonstrate that luteolin increases the levels of miR-132, which serves as an important regulator for neurite outgrowth in PC12 cells. Furthermore, we identify the involvement of the cAMP/PKA- and ERK-dependent CREB signaling pathways in the luteolin-mediated miR-132 expression and neuritogenesis of PC12 cells.

## Materials and Methods

### Chemicals

Luteolin, poly-L-lysine, dimethyl sulfoxide (DMSO), forskolin, KN-62 [1-[N,O-bis(5-isoquinolinesulfonyl)-N-methyl-L-tyrosyl]-4- phenylpiperazine], H-89 [N-[2-((*p*-Bromocinnamyl)amino)ethyl]-5-isoquinolinesulfonamide], 2-naphthol AS-E phosphate (KG-501), AG1478 and other chemicals were purchased from Sigma-Aldrich Co. (St. Louis, MO) unless otherwise indicated. U0126 [1,4-diamino-2,3-dicyano-1,4- bis (2-aminophenylthio)butadiene], a selective and potent inhibitor of MAPK/ERK kinase (MEK) activity, was purchased from Promega (Madison, WI, USA). Bisindolylmaleimide I (BIM), a protein kinase C inhibitor, was purchased from Cayman Chemical (Ann Arbor, MI, USA). SQ22536 [9-(Tetrehydro-2-furyl) adenine], a cell-permeable adenylate cyclase inhibitor, and TrkA antagonist K252a were purchased from Enzo Life Sciences (Ann Arbor, MI, USA).

### Cell Culture

PC12 cells, the rat adrenal pheochromocytoma cell line, were obtained from Bioresource Collection and Research Center (Hsinchu, Taiwan) and maintained in complete medium containing RPMI-1640 (Sigma-Aldrich), 2 mM glutamine, 1.5 g/L sodium bicarbonate, 4.5 g/L glucose, 10 mM HEPES and 1 mM sodium pyruvate and supplemented with 10% heat-inactivated horse serum (HS) (Invitrogen, Carlsbad, CA, USA) and 5% fetal bovine serum (FBS)(Biological Industries, Kibbutz Haemek, Israel) in a 5% CO2 incubator at 37°C.

### Analysis of Cell Viability by MTT Assay

Cell viability was measured by the mitochondrial-dependent reduction of 3-(4, 5-dimethylthiazol-2-yl)-2, 5-diphenyl tetrazolium bromide (MTT) to purple formazan. Briefly, cells were incubated with MTT solution (1 mg/ml final concentration) for 4 h at 37°C. The medium was carefully removed and formazan crystals were dissolved in dimethyl sulfoxide (DMSO). The extent of the reduction of MTT was determined by measurement of the absorbance at 550 nm.

### Analysis of Neurite Outgrowth in PC12 Cells

Before starting the experiment, 6-well culture plates were coated with poly-L-lysine as follows: 1 ml of a sterile aqueous solution of poly-L-lysine hydrobromide (0.1 mg/mL) was added to each well. After rocking gently to ensure coating of the well surface for 1 h, the solution was removed by aspiration. The surface of the dish was rinsed with sterile water and dried for at least 2 h before introducing the cells and medium.

The quantification of neurite outgrowth in PC12 cells was performed as described previously [14]. Briefly, cells (2×10^5^/ml, passage number <10) were seeded in poly-L-lysine-coated 6-well plates with normal serum medium, and after 24 h, the cells were changed to low serum (1% horse serum and 0.5% FBS) medium and treated with vehicle (0.1% DMSO) or the indicated reagents for 72 h. The PC12 cells were photographed by an inverted microscope (Olympus IX71) using phase-contrast objectives and examined later by counting the cells positive for neurites. The neurite-bearing cells were counted from at least ten randomly selected microscopic fields with an average of 100 cells per field. The number of differentiated cells was determined by a visual examination of the field and by counting those cells that had at least one neurite with a length equal to the cell body diameter; data are expressed as a percentage of the total cells in the field. The neurite length was also measured for all neurite-bearing cells identified in a field by tracing the longest neurite length per cell using Image J 1.42 software (NIH Image software). Each experiment was conducted in triplicate.

### Reverse Transcription Quantitative PCR (RT-Q-PCR) of miRNA

PC12 cells (1×10^6^/ml) were seeded on poly-L-lysine-coated 6-well plates in normal medium for 24 h. The cells were then shifted to low serum (1% HS and 0.5% FBS) as indicated for 24 h prior to exposure to vehicle (0.1% DMSO) or the indicated reagents for the indicated period. Cellular RNA was prepared using the Trizol reagent (Invitrogen) according to the manufacturer’s protocol. For the quantization of immature forms of miR-132 (pri- and pre-miR-132), 2 µg of total RNA was first reverse-transcribed using a High Capacity cDNA reverse transcription kit (Applied Biosystems, Foster City, CA, USA). Quantitative real-time PCR was then performed with 2 µL cDNA obtained above in 25 µL containing 200 nM primers [pre-miR-132, 5′-CCGCGTCTCCAGGGCAAC-3′ (forward) and 5′-CCTCCGGTTCCCACAGTAACAA-3′ (reverse) [10]; β-actin, 5′-CCTCTGAACCCTAAGGCCAA-3′ (forward) and 5′-AGCCTGGATGGCTACGTACA-3′ (reverse)] and Maxima SYBR Green/ROX qPCR Master Mix (Fermentas, Burlington, CA). Amplification was conducted in an ABI Prism 7300 Real-Time PCR System. The PCR conditions were as follows: 94°C for 4 min, 40 cycles at 94°C for 1 min, 58°C for 1 min, and 72°C for 1 min. The ΔΔC_t_ method was used for the data analysis of immature forms of miR-132 estimated in triplicate samples and normalized to β-actin expression levels.

For the quantification of mature form of miR-132, reverse transcription of 10 ng RNA was performed using a TaqMan MicroRNA Reverse Transcription kit (Applied Biosystems) followed by a TaqMan miRNA assay (Applied Biosystems) using primers and probes specific for miR-132 or for the U6 RNA internal control according to the manufacturer’s protocol. Amplification was conducted in an ABI Prism 7300 Real-Time PCR System. The PCR conditions were as follows: 95°C for 10 min, 40 cycles at 95°C for 15 sec, 60°C for 1 min. The ΔΔC_t_ method was used for the data analysis of mature form of miR-132 estimated in triplicate samples and normalized to U6 internal control expression levels.

### Transfection of miRNA Antisense, miRNA Mimics and Small Interference RNA (siRNA) Oligonucleotides

PC12 cells were seeded on poly-L-lysine-coated 6-well plates in RPMI-containing normal serum medium for 24 h. Cells were then shifted to serum-free OPTI-MEM medium and transfected with miR-132 antisense (Ambion Anti-miR™ miRNA inhibitor), scrambled control (Ambion Anti-miR™ negative control#1) (Applied Biosystems),miR-132 mimics (micrONTM rno-miR-132 mimics), mimics negative control (micrONTM mimics negative control#22) (Phalanx Biotech, Hsinchu, Taiwan), the 2′-OMe modified siRNA negative control, and rat-specific CREB siRNA duplexes [5′-GGAGUCUGUGGAUAGUGUA-3′ (forward) and 5′-UACACUAUCCACAGACUCC-3′ (reverse)] [58] (GeneDireX Inc., Las Vegas, NV, USA) at a final concentration of 150 pmol using the Lipofectamine 2000 reagent (Invitrogen) for 5 h according to the manufacturer’s instructions. Cells were then changed into RPMI normal serum medium. Twenty-four hours after transfection, the cells were changed to low-serum medium and treated with vehicle (0.1% DMSO) or luteolin (20 µM) for further indicated analysis.

### Western Blotting Analysis of CREB, Phospho-CREB, ERK, Phospho-ERK and β-actin Proteins

PC12 cells (1×10^6^/ml) were seeded in poly-L-lysine-coated 100 mm dishes in normal serum medium for 24 h and then shifted to low-serum medium (1% horse serum and 0.5% FBS) for 24 h prior to exposure to vehicle (0.1% DMSO) or the indicated reagents for the indicated periods. The cells were washed with PBS, scraped in ice-cold RIPA buffer (Thermo Fisher Scientific, Inc., Rockford, IL) and incubated on ice for 15 min. The cellular debris was removed by centrifugation (8,000×g for 15 min) at 4°C, and the cell lysate was carefully transferred to a microcentrifuge tube. The protein concentration was measured by the Bradford method (Bio-Rad Laboratories, Hercules, CA, USA) using bovine serum albumin as the standard.

Cell lysate (30 µg) was separated on 10% SDS-PAGE and transferred onto a PVDF membrane (PerkinElmer, Boston, MA, USA) at 25 volts overnight at 4°C. The membranes were blocked at 4°C in PBST blocking buffer (1% non-fat dried milk in PBS containing 0.1% Tween-20) for 8 h. Blots were incubated with the appropriate antibodies overnight at 4°C: anti-CREB (1∶1000), anti-phospho-CREB (Ser-133) (1∶1000), anti-ERK (1∶1000 ), anti-phospho-ERK (1∶1000 ) (Cell Signaling Technology, Inc.), and Monoclonal anti-β actin (1∶8000) (Sigma-Aldrich Co.). After three washes with PBST, the blots were incubated with the appropriate horseradish peroxidase-conjugated secondary antibodies (1∶10,000) (Santa Cruz Biotechnology, Santa Cruz, CA) for 1 h. The blots were washed with PBST and the proteins were detected by Western Lightning™ Chemiluminescence Reagent *Plus* (PerkinElmer) according to the manufacturer’s instructions, and the chemiluminescence signal was visualized with Amersham Hyperfilm™ ECL (GE Healthcare, Buckinghamshire, UK).

### Reporter Gene Assay for Cyclic AMP Response Element (CRE)-mediated Transcription Activity

PC12 cells (2×10^5^/well) were seeded on poly-L-lysine-coated 24-wells plate in DMEM containing 10% horse serum and 5% FBS medium for 24 h. For transient transfection, cells were co-transfected with the pCRE-Luc Cis-reporter plasmid (Stratagene, La Jolla, CA, USA) and *Renilla* luciferase vector (Promega) using Lipofectamine 2000 reagent (Invitrogen) for 4 h. Cells were then changed into DMEM supplemented with 20% horse serum and 10% FBS. Twenty-four hours after transfection, the cells were changed to low-serum medium and treated with vehicle (0.1% DMSO) or luteolin (20 µM) for 8 h and harvested by using Passive Lysis Buffer (Promega). Luciferase activities were determined by the Dual-Luciferase Reporter Assay System Kit (Promega) according to the manufacturer’s instructions. The intensities of the luciferase reactions measured in the lysates of the transfectants were normalized to their activity of *Renilla* luciferase, which was used as an internal control.

### Analysis of Protein Kinase A (PKA) Activity by Enzyme Immunoassay

Protein kinase A (PKA) activity was analyzed by a nonradioactive PKA Activity Assay Kit (EKS-390A, Enzo Life Sciences). PC12 cells (1×10^6^/ml ) were seeded on poly-L-lysine-coated 100 mm dishes in normal serum medium for 24 h, then shifted to low serum (1% HS and 0.5% FBS) as indicated for 24 h prior to exposure to luteolin for indicated periods. Cellular proteins were collected using lysis buffer according to the manufacturer’s instruction. PKA substrate microtiter plate, which was pre-coated with PKA substrate, was soaked with kinase assay dilution buffer for 10 min at room temperature. 30 µl of cell lysates (0.1 µg) or PKA standard (0.01 µg) were then added, followed by the addition of ATP to initiate the reaction. After incubation at 30°C for 90 min, the reaction mixture was removed from the plate, and phosphospecific substrate antibody was added to each well and incubated at room temperature for 60 min. The liquid was aspirated and wells were repeatedly washed. HRP-conjugated secondary anti-rabbit IgG was then added to each well and incubated for another 30 min at room temperature. The wash was repeated after incubation and TMB substrate solution was added to each well. Stop solution was added after 30–60 min and the 96-well plate was read at 450 nm in a microplate reader.

### Statistical Analysis

All experiments were repeated at least three times. All values are expressed as the mean ± SD. The results were analyzed by Student’s unpaired *t*-test, and a *p* value of <0.05 was taken to be significant.

## Supporting Information

Figure S1 **Chemical structure of luteolin (3′,4′,5,7-tetrahydroxyflavone).**
(TIF)

Figure S2 **Effects of luteolin on the cell viability of PC12 cells.** PC12 cells were seeded on poly-L-lysine-coated 6-well plates in normal-serum medium for 24 h. Cells were then shifted to low-serum medium (1% horse serum and 0.5% FBS) for 24 h prior to exposure to vehicle (0.1% DMSO) or luteolin (20 µM) for additional 24 h. Cell viability was determined by MTT assay as described in the Materials and Methods and expressed as percentage of control group, which represents the cell counts prior to medium change. Data represent the mean ± SD from three independent experiments. **p<0.01 represent significant differences compared with vehicle group cells.
(TIF)

Figure S3 **Effects of miR-132 antisense on the cell viability and expression of miR-132 in PC12 cells.** PC12 cells were seeded on poly-L-lysine-coated 6-well plates in normal serum medium for 24 h. The cells were then transfected with miR-132 antisense oligonucleotides (anti-miR-132) or a scramble antisense negative control (anti-NC) for 24 h as described in Materials and Methods. After transfection, PC12 cells were shifted to low-serum medium (1% horse serum and 0.5% FBS) and exposed to vehicle (0.1% DMSO) or luteolin (20 µM). **(A)** Cell viability was determined by MTT assay as described in the Materials and Methods. **(B)** After 2 h treatment, the cellular RNA was then prepared and the levels of mature miR-132 were detected by reverse transcription quantitative PCR as described in Materials and Methods. Data represent the mean ± SD from three independent experiments. **p<0.01 represents significant differences compared to vehicle-treated cells. ##*p*<0.01 represents significant differences compared to antisense-untreated cells.
(TIF)

Figure S4 **Effect of miR-132 over-expression on the neurite outgrowth in PC12 cells.** PC12 cells were seeded on poly-L-lysine-coated 6-well plates in normal serum medium for 24 h. The cells were then transfected with miR-132 mimics (miR-132; 100 pmol and 150 pmol) or miRNA negative control (miR-NC; 100 pmol and 150 pmol) for 24 h as described in Materials and Methods. After transfection, PC12 cells were shifted to low-serum medium (1% horse serum and 0.5% FBS) and exposed to vehicle (0.1% DMSO) for an additional 72 h. Neurite-bearing cells were analyzed as described in Materials and Methods. Data represent the mean ± SD from three independent experiments. **p<0.01 represents significant differences compared to miR-NC transfected group.
(TIF)

Figure S5 **KG-501 inhibits CRE-mediated transcription activity in luteolin-treated cells.** PC12 cells were seeded on poly-L-lysine-coated 24-well plates in DMEM containing 10% horse serum and 5% FBS for 24 h. Cells were then transfected with a CRE-mediated luciferase reporter construct and *Renilla* luciferase control plasmid for 24 h. After transfection, cells were pre-treated with inhibitor KG-501 (10 µM) for 30 min and then exposed to vehicle (0.1% DMSO) or luteolin (20 µM) for an additional 8 h. The intensities of the luciferase reactions measured in the lysates of the transfectants were normalized to their *Renilla* luciferase control activity. Data represent the mean ± SD from three independent experiments. **p<0.01 represents significant differences compared to KG-501-untreated cells.
(TIF)
