# Supplementary figures and images for: Luteolin Induces microRNA-132 Expression and Modulates Neurite Outgrowth in PC12 Cells
Source: PLoS One. 2012 Aug 16;7(8):e43304. doi: 10.1371/journal.pone.0043304 (PMC3420912; doi:10.1371/journal.pone.0043304)

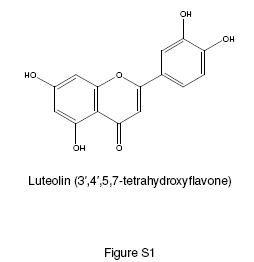

Supplement: Figure S1 — Chemical structure of luteolin (3′,4′,5,7-tetrahydroxyflavone). (TIF) [file pone.0043304.s001.tif]

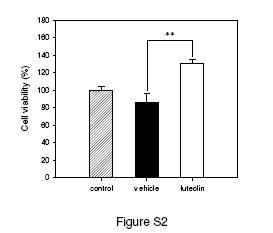

Supplement: Figure S2 — Effects of luteolin on the cell viability of PC12 cells. PC12 cells were seeded on poly-L-lysine-coated 6-well plates in normal-serum medium for 24 h. Cells were then shifted to low-serum medium (1% horse serum and 0.5% FBS) for 24 h prior to exposure to vehicle (0.1% DMSO) or luteolin (20 µM) for additional 24 h. Cell viability was determined by MTT assay as described in the Materials and Methods and expressed as percentage of control group, which represents the cell counts prior to medium change. Data represent the mean ± SD from three independent experiments. **p<0.01 represent significant differences compared with vehicle group cells. (TIF) [file pone.0043304.s002.tif]

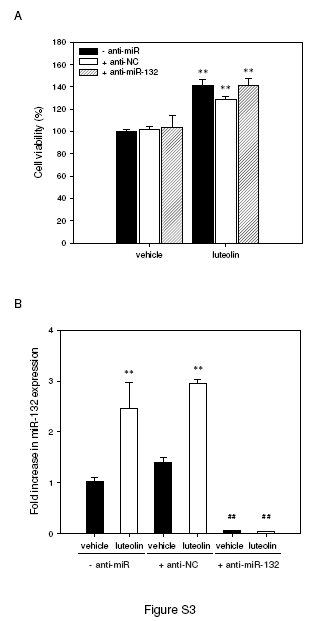

Supplement: Figure S3 — Effects of miR-132 antisense on the cell viability and expression of miR-132 in PC12 cells. PC12 cells were seeded on poly-L-lysine-coated 6-well plates in normal serum medium for 24 h. The cells were then transfected with miR-132 antisense oligonucleotides (anti-miR-132) or a scramble antisense negative control (anti-NC) for 24 h as described in Materials and Methods. After transfection, PC12 cells were shifted to low-serum medium (1% horse serum and 0.5% FBS) and exposed to vehicle (0.1% DMSO) or luteolin (20 µM). (A) Cell viability was determined by MTT assay as described in the Materials and Methods. (B) After 2 h treatment, the cellular RNA was then prepared and the levels of mature miR-132 were detected by reverse transcription quantitative PCR as described in Materials and Methods. Data represent the mean ± SD from three independent experiments. **p<0.01 represents significant differences compared to vehicle-treated cells. ##p<0.01 represents significant differences compared to antisense-untreated cells. (TIF) [file pone.0043304.s003.tif]

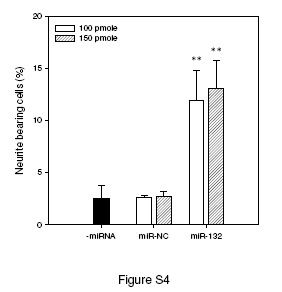

Supplement: Figure S4 — Effect of miR-132 over-expression on the neurite outgrowth in PC12 cells. PC12 cells were seeded on poly-L-lysine-coated 6-well plates in normal serum medium for 24 h. The cells were then transfected with miR-132 mimics (miR-132; 100 pmol and 150 pmol) or miRNA negative control (miR-NC; 100 pmol and 150 pmol) for 24 h as described in Materials and Methods. After transfection, PC12 cells were shifted to low-serum medium (1% horse serum and 0.5% FBS) and exposed to vehicle (0.1% DMSO) for an additional 72 h. Neurite-bearing cells were analyzed as described in Materials and Methods. Data represent the mean ± SD from three independent experiments. **p<0.01 represents significant differences compared to miR-NC transfected group. (TIF) [file pone.0043304.s004.tif]

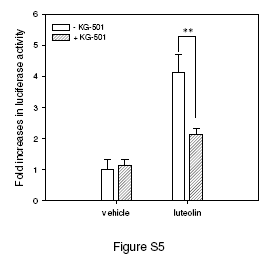

Supplement: Figure S5 — KG-501 inhibits CRE-mediated transcription activity in luteolin-treated cells. PC12 cells were seeded on poly-L-lysine-coated 24-well plates in DMEM containing 10% horse serum and 5% FBS for 24 h. Cells were then transfected with a CRE-mediated luciferase reporter construct and Renilla luciferase control plasmid for 24 h. After transfection, cells were pre-treated with inhibitor KG-501 (10 µM) for 30 min and then exposed to vehicle (0.1% DMSO) or luteolin (20 µM) for an additional 8 h. The intensities of the luciferase reactions measured in the lysates of the transfectants were normalized to their Renilla luciferase control activity. Data represent the mean ± SD from three independent experiments. **p<0.01 represents significant differences compared to KG-501-untreated cells. (TIF) [file pone.0043304.s005.tif]
